# System analysis based on the T cell exhaustion‑related genes identifies CD38 as a novel therapy target for ovarian cancer

**DOI:** 10.32604/or.2023.029282

**Published:** 2023-06-27

**Authors:** TIANMING SHI, RONGRONG YAN, MI HAN

**Affiliations:** 1 Department of Gynecologic Oncology, The International Peace Maternity and Child Health Hospital, School of Medicine, Shanghai Jiao Tong University, Shanghai, China; 2 Shanghai Key Laboratory of Embryo Original Diseases, School of Medicine, Shanghai Jiao Tong University, Shanghai, China

**Keywords:** CD8+ T exhausted, Ovarian cancer, Prognostic model, Single cell sequencing

## Abstract

Ovarian cancer (OV) is highly heterogeneous tumor with a very poor prognosis. Studies increasingly show that T cell exhaustion is prognostically relevant in OV. The aim of this study was to dissect the heterogeneity of T cell subclusters in OV through single cell transcriptomic analysis. The single RNA-sequencing (scRNA-seq) data of five OV patients were analyzed, and six major cell clusters were identified after threshold screening. Further clustering of T cell-associated clusters revealed four subtypes. Pathways related to oxidative phosphorylation, G2M checkpoint, JAK-STAT and MAPK signaling were significantly activated, while the p53 pathway was inhibited in the CD8+ exhausted T cells. The standard marker genes of CD8+ T cell exhaustion were screened to develop a T-cell related gene score (TRS) based on random forest plots in TCGA cohort. The patients with low TRS have better prognosis compared to the patients with high TRS in both TCGA and GEO. In addition, most genes included in the TRS showed significant differences in expression levels between the high- and low-risk groups. Immune cell infiltration was analyzed using the MCPcounter and xCell algorithms, which revealed significant differences between the two risk groups, indicating that the different prognoses may stem from the respective immune landscapes. In addition, CD38 knockdown in OV cell lines increased apoptosis and inhibited invasion *in vitro*. Finally, we performed a drug sensitivity analysis and identified six potential drug candidates for OV. To summarize, we identified the heterogeneity and clinical significance of T cell exhaustion in OV and built a superior prognostic model based on T cell exhaustion genes, which can contribute to the development of more precise and effective therapies.

## Introduction

Ovarian cancer (OV) is one of the deadliest and most aggressive cancers in women, and the incidence has increased in recent years [1]. Since the early symptoms of OV are insidious and the cancer develops rapidly, most patients are not diagnosed until the late stage of the disease [2]. Furthermore, the heterogenous nature of ovarian tumors restricts the predictive accuracy of existing prognostic markers, which also worsens patient prognosis [3]. Currently, surgery and chemoradiotherapy are the most common treatment strategies for OV. However, the considerable adverse effects of these approaches severely reduce the patients’ quality of life [4]. Given that the heterogeneity of ovarian tumors is a key factor affecting cancer progression and overall survival [5], and results in different responses of individual patients to the same treatment [6], it is crucial to use single-cell technologies to identify effective prognostic biomarkers [7,8]. In fact, analyses of tumors at the single-cell level have provided new insights into the molecular mechanisms underlying carcinogenesis, and revealed novel therapeutic possibilities [9–13]. A recent study examined the heterogeneity of OV by single-cell RNA-sequencing (scRNA-seq) [14]. In addition, Hu et al. were able to predict cancer behavior based on the analysis of individual fallopian tube epithelial cells [15]. Therefore, scRNA-seq studies can also improve our understanding of OV.

In recent years, immunotherapy has emerged as one of the most promising modalities of cancer treatment [16–18]. The tumor-infiltrating CD8+ T cells, the main effector cells of anti-cancer immunity [19,20], show impaired viability and proliferation, increased apoptosis, decreased production of effector cytokines, and lower cell killing ability [21]. Studies show that the majority of the anti-tumor CD8+ T cells are in the exhausted state [22], and do not express interleukin-2 (IL-2) or tumor necrosis factor α (TNF-α) [22,23]. CD8+ T cell exhaustion is a significant prognostic factor in liver cancer and colorectal cancer [24,25]. However, the prognostic relevance of CD8+ T cell exhaustion in OV has not been established so far.

In this study, we analyzed the single-cell transcriptional profiles obtained from OV patients, and identified four T cell-associated subtypes. The signature genes of the exhausted CD8+ T cell subset were screened to establish the T-cell related gene score (TRS) by random forest plot method. Based in the TRS, the patients were divided into the low- and high-risk groups, which differed in terms of prognosis, immune cell infiltration and drug sensitivity. Furthermore, the role of CD38, one of the signature genes in the TRS, in the malignant potential of OV cells was verified through *in vitro* experiments.

This study provides a more comprehensive understanding of the heterogeneity of T cells in ovarian tumors and the clinical significance of T cell exhaustion in OV. The prognostic model based on the TRS can help develop more precise and effective therapies.

## Materials and Methods

### Data collection

The single-cell sequencing data of 5 OV patients (GSE154600) was retrieved from the Gene Expression Omnibus (GEO, https://www.ncbi.nlm.nih.gov/geo/) database. The RNA-seq data and accompanying clinical information of 378 OV patients were downloaded from The Cancer Genome Atlas (TCGA, https://portal.gdc.cancer.gov/). In addition, the GSE140082 dataset including the RNA-seq and clinical data of 379 OV patients was also downloaded from the GEO database as the validation cohort. All available datasets used in this study were based on published reports that had received ethical approval. Dataset inclusion criteria: (1) Pathological diagnosis of ovarian cancer; (2) Complete transcriptome and clinical prognostic data; Exclusion criteria: (1) The pathological diagnosis was unclear; (2) Survival time less than 1 day; (3) The transcriptome information did not contain T cell exhaustion related genes.

### Data filtering and correction

The scRNA-seq data was analyzed using the “Seurat”, “SingleR” software packages. The cells with unique feature counts >5000 or <200, or with mitochondrial counts >5% were filtered out. The data of each retained cell was normalized using the default parameters of Seurat’s ‘NormalizeData’ function on feature-expression measurements, and then transferred to a combined Seurat object via the Harmony package. The variable genes were scaled and subjected to principal component analysis (PCA). The significant principal components (PCs) were selected by the “RunUMAP” function (min. dist = 0.2, n. neighbors = 20) and the “FindClusters” function (resolution = 0.5) for umap analysis and cluster analysis.

### Cell annotation

The individual cell types were annotated by two modalities. The T-cell subsets were initially clustered using SingleR, an automated annotation method for scRNA-seq data [26], and the identity of each cluster was further determined by manually searching the cell markers (http://biocc.hrbmu.edu.cn/CellMarker/). The differentially expressed genes (DEGs) in the CD8+ exhausted T cell subset were screened using the FindAllMarker function in the R “Seurat” package, with logFC > 0.3 and *p* < 0.05 as the thresholds.

### Enriched pathway analysis

The differences in the biological functions of the T cell-related subsets were determined by gene set enrichment analysis (GSEA), gene ontology (GO) and KEGG pathway analyses using R ‘ABGSEase’ and ‘clusterprofiler’ packages with Hallmark, GO and KEGG reference gene sets. Maximum gene set size (maxGSSize) was set to 100, minimum gene set size (minGSSize) to 50, and *p*-value < 0.05 was considered statistically significant.

### Construction of prognostic model

The DEGs in the CD8+ exhausted T cell subsets in the TCGA and GEO cohorts were corrected to the same level, and the prognostic genes were identified through univariate Cox regression analysis (*p* < 0.01). A random forest model was then built using the R package “randomForest”. The mean model inaccuracies for all genes were first estimated. The optimal number of trees in the random forest model was set at 500, and the dimensional effect sizes of the model were determined using the Gini coefficient method. Genes with importance indices higher than 0.6 were selected as disease hub genes. TRS was calculated using the following formula:

$$\begin{array}{l} TRS=\;\;Coef_{1}\times Geneexpression_{1}+Coef_{2}\times Geneexpression_{2}\\ \;+\cdots Coef_{n}\times Geneexpression_{n} \end{array}$$

The Cox regression coefficient represents the prognostic value of each gene. Expression values for genes correspond to those of their modeled counterparts. Based on the median TRS, OV patients were classified as high-risk or low-risk. The R “survival” package [27,28] was used to plot Kaplan-Meier survival curves. Based on clinical features, the “rms” package of R software was used to develop a nomogram to predict survival of individual patients [29].

### Immune cell infiltration analysis

The infiltration of different immune cell populations was quantified based on the transcriptional profiles of patients using MCPcounter [30] and xCell [31] algorithms. In addition, the absolute content of the four T cell subsets were derived from the reference single-cell dataset. The data was visualized using boxplots, heat maps, and scatter plots.

### Drug sensitivity analysis

The sensitivity score of the drugs in the Genomics of Drug Sensitivity in Cancer (GDSC) database was calculated with the R package ‘oncoPredict’ [32]. Wilcox’s test was used to identify the candidate drugs with *p* < 0.05 as the threshold for statistical significance.

### Cell culture

The A2780 OV cell line and IOSE80 normal ovarian cell line were obtained from the American Type Culture Collection (ATCC, Rockville, MD, USA). The cells were cultured in RPMI-1640 medium supplemented with 10% FBS at 37°C and 5% CO_2_.

### Quantitative real-time PCR (qRT-PCR)

RNA was extracted from the cells using TRIzol reagent (Takara, Japan) and reverse transcribed into cDNA using SuperScript II Reverse Transcriptase (Invitrogen, USA), The relative expression level of CD38 was analyzed by qRT-PCR and normalized to GAPDH. The primers are listed in Suppl. Table S1.

### Cell transfection

The CD38 siRNA was mixed with the RNAiMAX transfection reagent in Opti-MEM for 20 min at room temperature, and then transfected into the OV cells. After 12 h, the medium was discarded and RPMI-1640 medium was added. The procedure was repeated after 12 h. The CD38-siRNA target sequences were as follows: sense 5′-GCCUGAUCUAUACUCAAAUTT-3′ and antisense 5′-AUUUGAGUAUAGAUCAGGCTT-3′. CD38 knockdown was verified by qRT-PCR.

### Apoptosis analysis

The transfected cells were washed with PBS, and harvested with trypsin digestion solution containing no EDTA (Solarbio, China). After centrifuging at 1000 rpm for 5 min, the cells were stained with 7-AAD (BD Biosciences, USA) and annexin-APC (BD Biosciences, USA) for 15 min.

### Cell invasion assay

The transfected cells were seeded on matrix gel-coated upper chamber of Transwell inserts in serum-free RPMI-1640 medium, and the lower chambers were filled with complete medium. After culturing for 24 h, the cells remaining on the upper membrane surface were removed with cotton swabs, and those that had adhered to the bottom surface were fixed with 4% paraformaldehyde and then stained with crystal violet. The cells were observed under a light microscope, and counted using ImageJ.

### Statistical analysis

R (version 4.1.0) program was used for statistical analyses. Overall survival was evaluated using Kaplan-Meier (KM) and log-rank tests, and differences among subgroups were determined with Wilcox test and Kruskal test.

## Results

### Cell clustering analysis of OV

**Figure 1:**
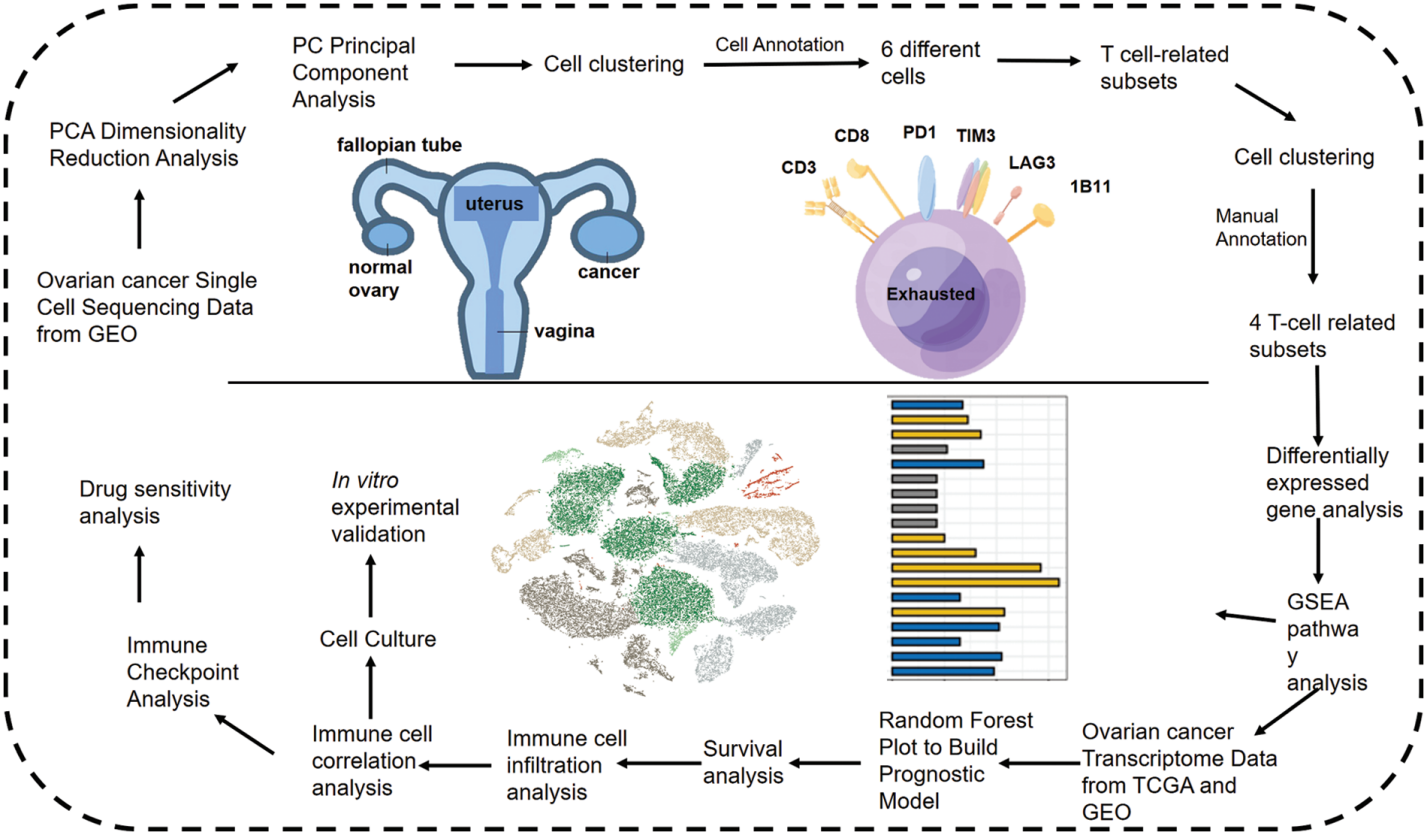
Flow chart shows the study design.

**Figure 2:**
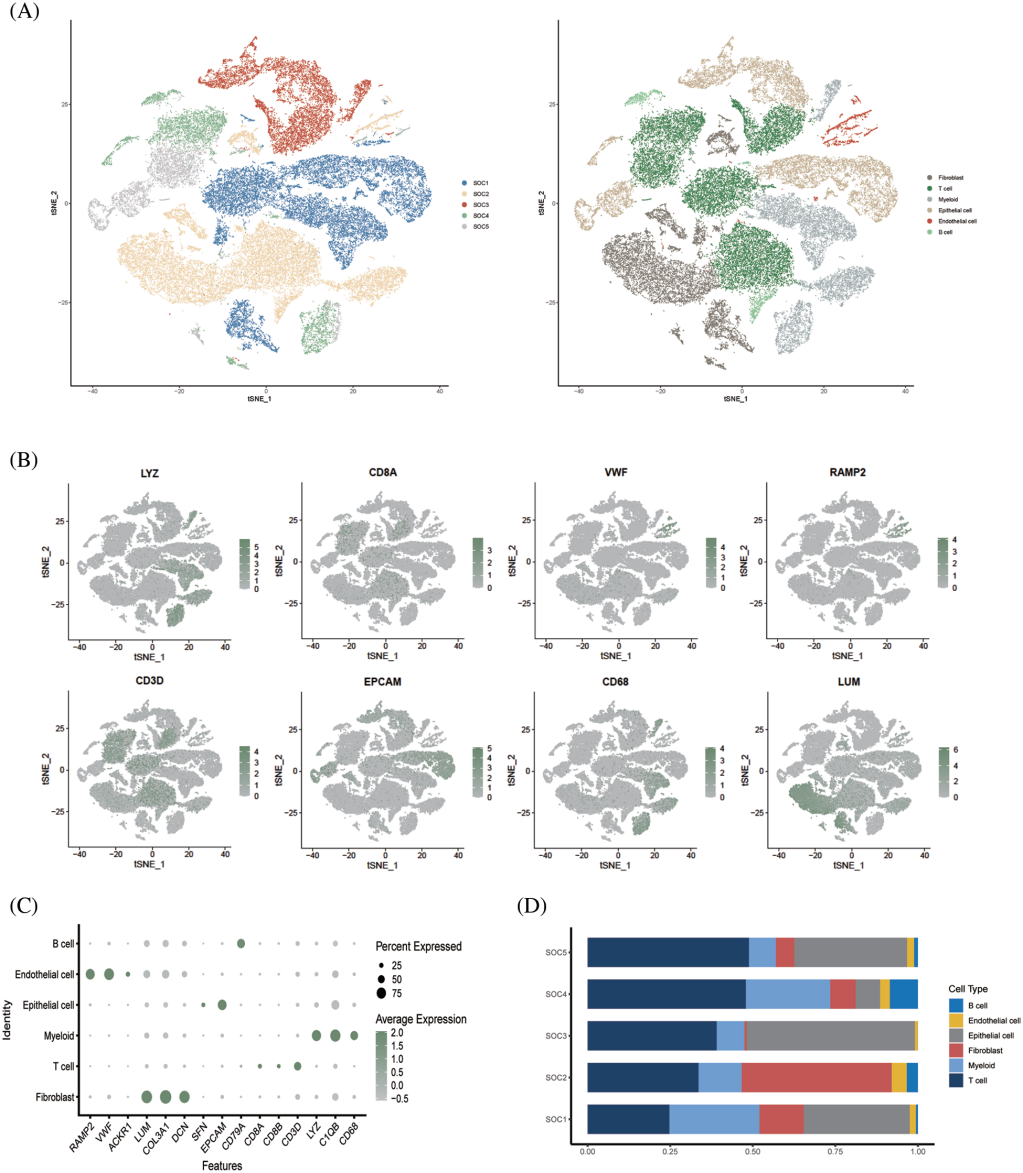
Single cell cluster analysis of OV. (A) The tSNE plots show a dimensionality reduction cluster analysis of single cells from five OV samples annotated as a total of six cells. (B) The tSNE plot shows the expression of signature genes in single-cell clusters. (C) Bubble plots showing expression of signature genes. (D) Histogram showing the amount of each cell type in the sample.

The study design is outlined in the flow chart in Fig. 1. The scRNA-seq data of obtained from 5 OV patients was screened, and the cells were annotated in terms of spatial distribution (Fig. 2A, left), as well as characteristic markers. Six cell types were annotated, including fibroblasts, T cells, myeloid cells, epithelial cells, endothelial cells, and B cells (Fig. 2A, right). The expression profiles of the eight classical marker genes are shown in Fig. 2B, and their expression in each cell type is shown in the bubble plots in Fig. 2C. Based on the absolute counts, the T cell subsets were predominant in almost all samples, suggesting a crucial role of these cells in OV progression (Fig. 2D). In addition, the content of the other cell types in each cell subset was significantly different, which confirms the cellular heterogeneity of OV.

### Dimensional cluster analysis of T cell subsets

**Figure 3:**
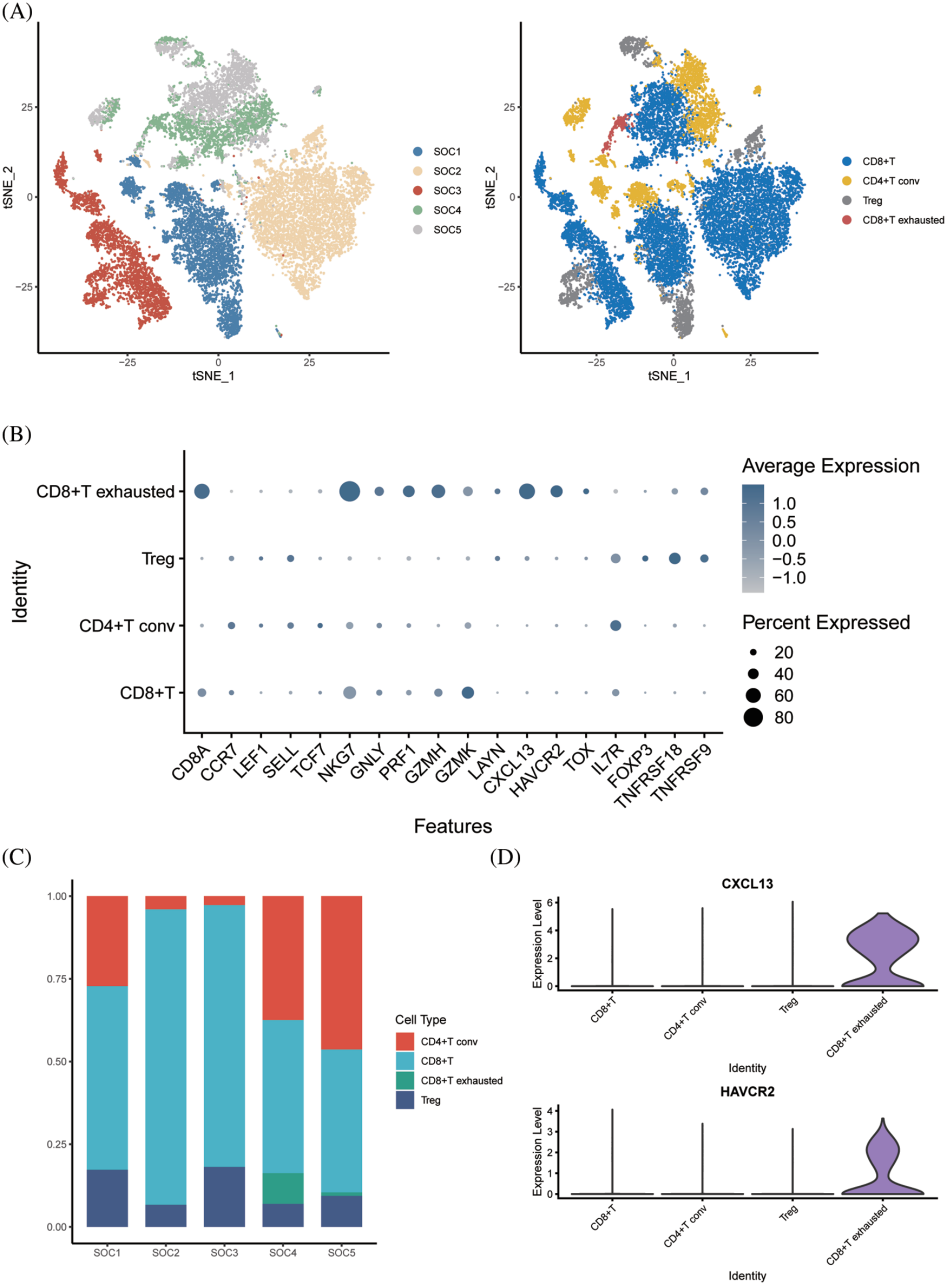
Dimensional cluster analysis of T cell subsets. (A) The tSNE plot shows the dimensionality reduction clustering of T cell subsets, annotated as a total of four T cell-associated subsets. (B) Bubble plots showing expression of signature genes. (C) Histogram showing the content of each T-cell related subset in the sample. (D) Violin plot showing expression of two signature genes in CD8+ exhausted T cells.

Following dimensionality reduction cluster analysis of T cell subsets, we annotated four subsets, including CD8+ T cells, CD4+ T conv cells, regulatory T cells (Tregs) and CD8+ exhausted T cells (Fig. 3A). The expression of signature genes in each cell subset is shown in the bubble plots in Fig. 3B. The CD8+ T cells were the most abundant subset among the samples (Fig. 3C), whereas the CD8+ exhausted T cells were the least abundant in each sample. Analysis of the differentially expressed genes in the T cell subsets indicated that *CXCL13* and *HAVCR2* were significantly upregulated in the CD8+ exhausted T cells (Fig. 3D).

### Enriched pathway analysis

**Figure 4:**
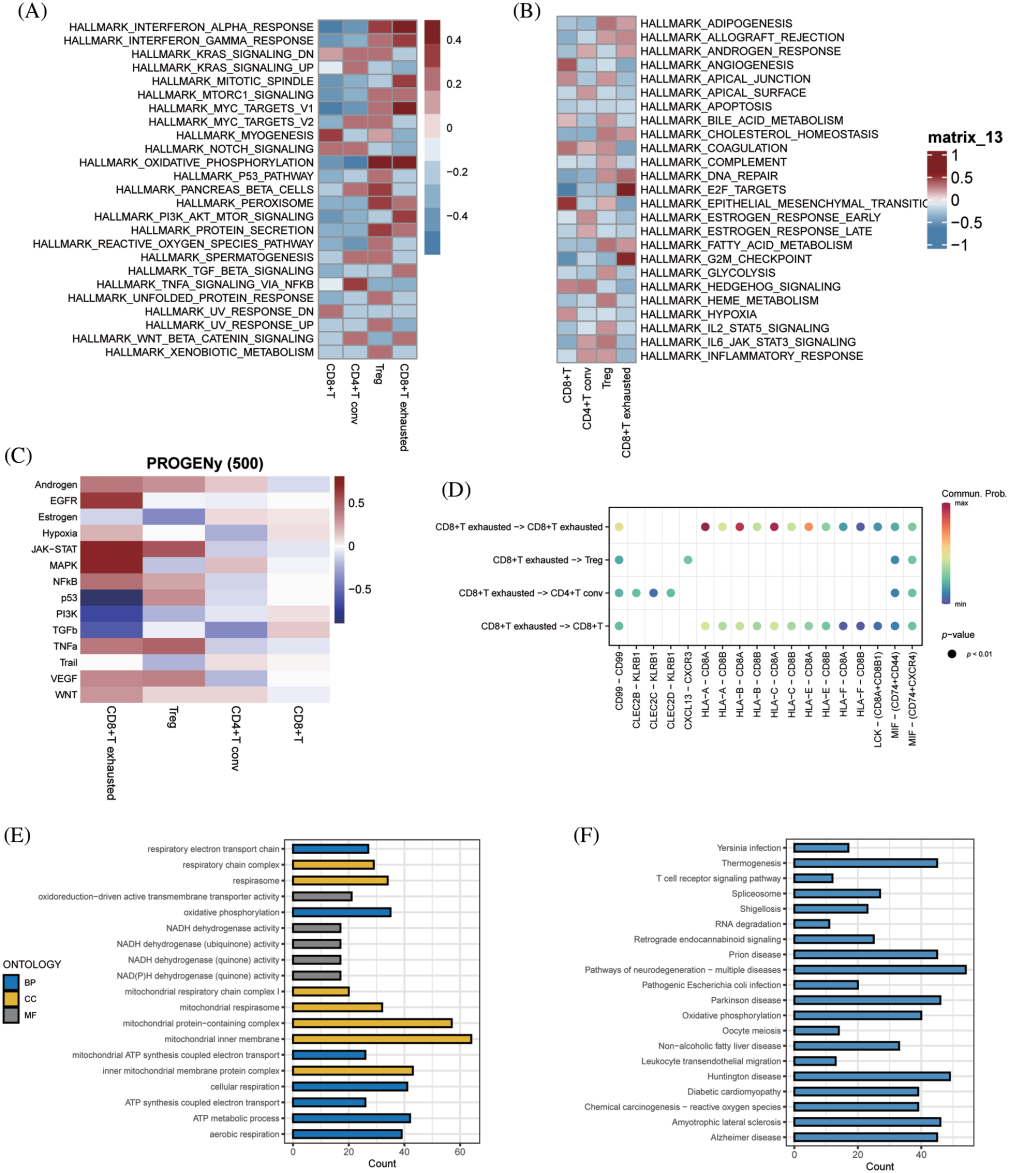
Enriched pathway analysis. (A, B) Heat map showing the expression of 50 hallmark tumor-associated pathways in each T cell subset. (C) Heat map showing the expression of 14 key pathways in each T cell subset. (D) Bubble plots showing CD8+ T-cell exhaustion *vs*. expression of ligand pairs for other cell subsets receptors. (E) Histogram of GO enriched pathways for DEGs in the CD8+ exhausted T cells. (F) Histogram of KEGG enriched pathways for the DEGs.

GSEA of tumor-associated pathways was performed to further explore the differences in the biological functions of the T cell subsets. The myogenesis and epithelial mesenchymal transition pathways were significantly activated in CD8+ T cells, and TNFA signaling via NFKB pathway in the CD4+ T conv cells. The Tregs were significantly associated with the oxidative phosphorylation, pancreatic beta cells and peroxidase pathways, whereas the CD8+ exhausted T cells showed significant enrichment of the interferon alpha response, interferon gamma response, MYC target V1, oxidative phosphorylation, E2F target and G2M checkpoint pathways (Figs. 4A and 4B). Subsequently, we analyzed tumor-associated pathways in different cell populations based on PROGENy score. As shown in Fig. 4C, The JAK-STAT and MAPK pathways were activated, and the p53 and PI3K pathways were inhibited in the CD8+ exhausted T cells. Receptor ligand pair analysis between CD8+ exhausted T cells and other T cell subsets revealed high enrichment of the HLA−A−CD8A, HLA−B−CD8A, and HLA−C−CD8A pairs (Fig. 4D). Given the central role of HLA molecules in immunity, our findings indicate that this subset may influence the response to immunotherapy. Furthermore, GO analysis of the DEGs between CD8+ exhausted T cells and controls showed significant enrichment of ATP metabolic process, aerobic respiration, cellular respiration and oxidative phosphorylation in biological processes (BP), redox-driven activity transmembrane transporter activity and NADH dehydrogenase activity in molecular function (MF), and mitochondrial inner membrane, mitochondrial protein complex and mitochondrial inner membrane protein complex in cellular compartment (CC) terms (Fig. 4E). Finally, the KEGG pathways significantly associated with the DEGs included those related to Huntington’s disease, prion disease, thermogenesis, amyotrophic lateral sclerosis, and other neurodegenerative diseases (Fig. 4F).

### Prognostic model for OV

**Figure 5:**
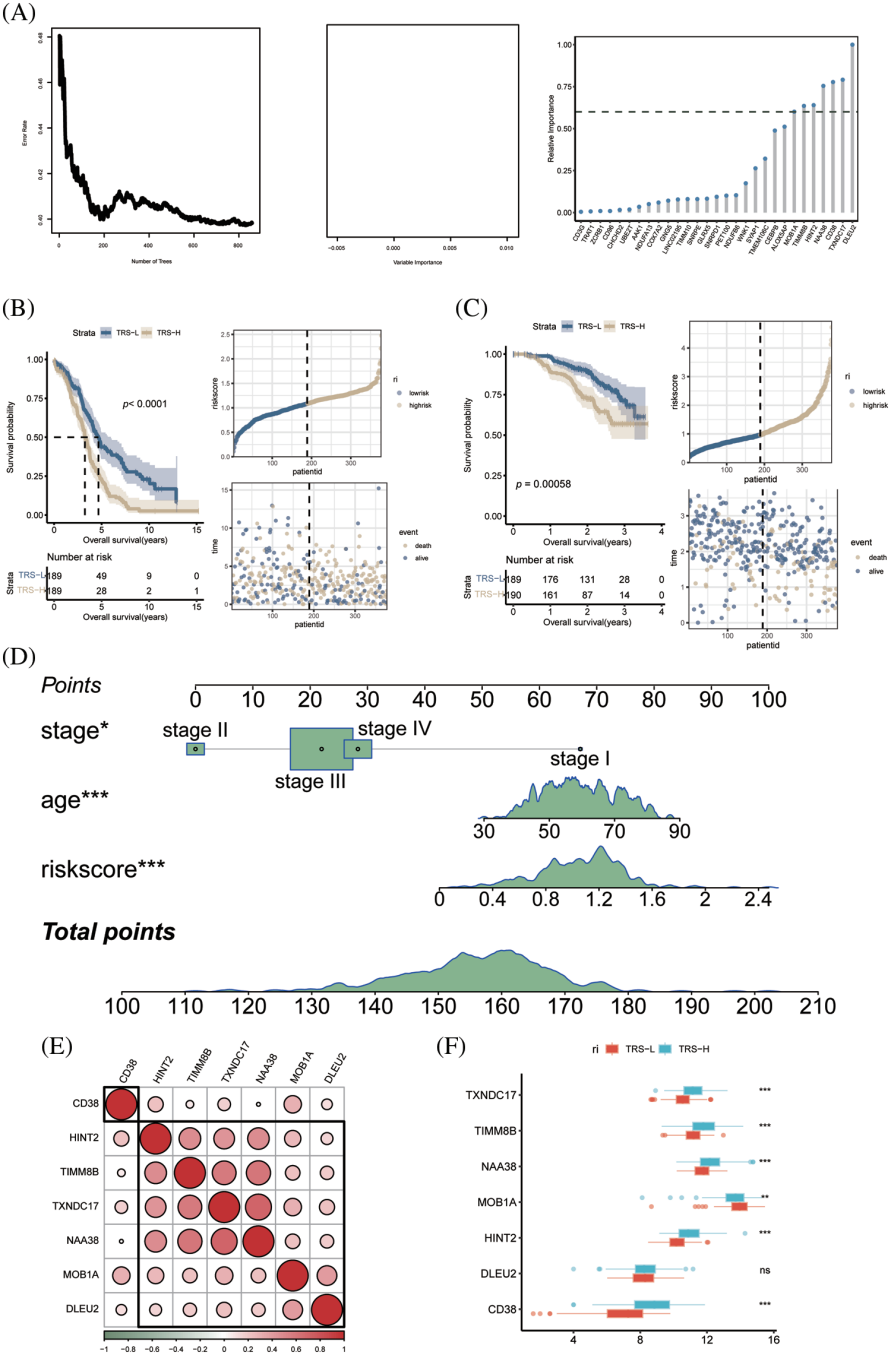
Construction of prognostic model of OV. (A) The number of selected trees impacts the error rate. The x-axis represents the number of decision trees, and the y-axis represents the error rate. Random forest classifier results based on Gini coefficient. The x-axis represents genetic variables and the y-axis represents importance indices. Seven genes with prognostic significance were selected from 47 genes based on random forest and prognostic models were constructed. (B) KM survival curves and scatter plot of survival distributions for the TCGA cohort. (C) KM survival curves and scatter plot for the GEO validation cohort. (D) Nomogram of OV prognostic model based on stage, age and TRS. (E) Pie charts show the correlation between each modeled gene. (F) Box plots showing the expression of each modeled gene between high- and low-risk groups.

To identify the prognostic genes associated with CD8+ T cell exhaustion, we performed univariate Cox regression analysis of the DEGs in this subset. We screened 47 prognostic genes that were incorporated into a random forest classifier. Seven genes, including *MOB1A*, *TIMM8B*, *CD38*, *HINT2*, *NAA38*, *DLEU2* and *TXNDC17*, were finally identified and prognostic models were constructed using a random forest classifier (Fig. 5A). The TRS was calculated as follows:

$$\begin{array}{l} TRS=\;\;0.25516\times MOB1A+\left(-0.07000\right)\times TIMM8B\\ \;+\left(-0.12049\right)\times HINT2+\left(-0.06368\right)\times NAA38\\ \;+\left(-0.15835\right)\times CD38+\left(-0.04413\right)\times TXNDC17\\ \;+0.04650\times DLEU2 \end{array}$$

According to the median risk score, the patients in TCGA cohort were divided into the low-risk and high-risk groups. As shown in Fig. 5B, the patients with low-risk score had significantly better OS. Using the median TRS of the training set, we classified patients in the external GSE140082 set as high- or low-risk. As expected, the low-risk group had significantly better OS compared to the high-risk group, thus validating our prognostic model (Fig. 5C). To accurately estimate the survival status of individual OV patients, we developed a predictive nomogram based on TRS, age and tumor stage. As shown in Fig. 5D, the TRS-based nomogram can accurately predict the survival of OV patients.

### Differentiation of high-risk and low-risk patients based on immune landscape

Except for *CD38*, the other genes in the prognostic model showed significant correlation with each other (Fig. 5E). In addition, *TIMM8B*, *CD38*, *HINT2*, *NAA38* and *TXNDC17* were significantly upregulated in the low-risk patients, whereas higher levels of *MOB1A* were detected in the high-risk group (Fig. 5F).

**Figure 6:**
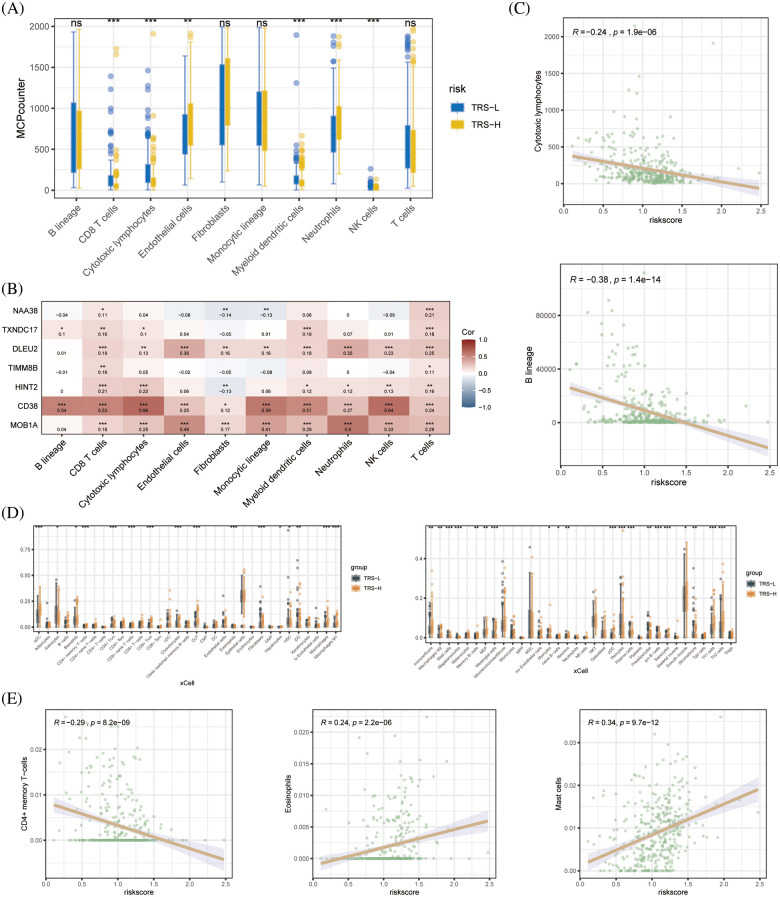
Immune cell infiltration analysis. (A) Boxplots showing differences in the infiltration of each cell subset in the high- and low-risk groups based on based on MCPcounter algorithm. (B) Heat map showing the correlation between each modeled gene and cell subset. (C) Correlation between the two cell types based on MCPcounter algorithm. (D) Box plots showing differences in the infiltration of each cell subset in the high- and low-risk groups based on the xCell algorithm. (E) Correlation between the three cell types based on the results of immune cell infiltration analysis by xCell algorithm.

Since the immune landscape plays a key role in tumor development and progression, we compared the immune infiltration in the high-risk and low-risk groups using MCPcounter and xCell. According to the MCPcounter algorithm, patients in the low-risk group had significantly higher number of infiltrating CD8+ T cells, cytotoxic lymphocytes, myeloid dendritic cells, and NK cells compared to patients in the high-risk group, whereas the latter had significantly higher abundance of neutrophils (Fig. 6A). We also detected a significant association between CD38 and most immune-related genes (Fig. 6B). In addition, the B lineage cells and cytotoxic lymphocytes showed a significant negative correlation with the TRS (Fig. 6C). The xCell algorithm also indicated significant differences between the immune cell composition of the high- and low-risk groups. As shown in Fig. 6D, the aDC cells, M1 macrophages, M2 macrophages, Th1 cells and Th2 cells were significantly more abundant in the high-risk *vs*. the low-risk patients. Therefore, the poor prognosis in the high-risk group may be due to the distinct immune landscape. In addition, we found a significant negative correlation between CD4+ memory T cells and TRS, and a significant positive correlation between eosinophils and mast cells and TRS (Fig. 6E).

### CD38 knockdown in OV cells inhibited their malignant potential

**Figure 7:**
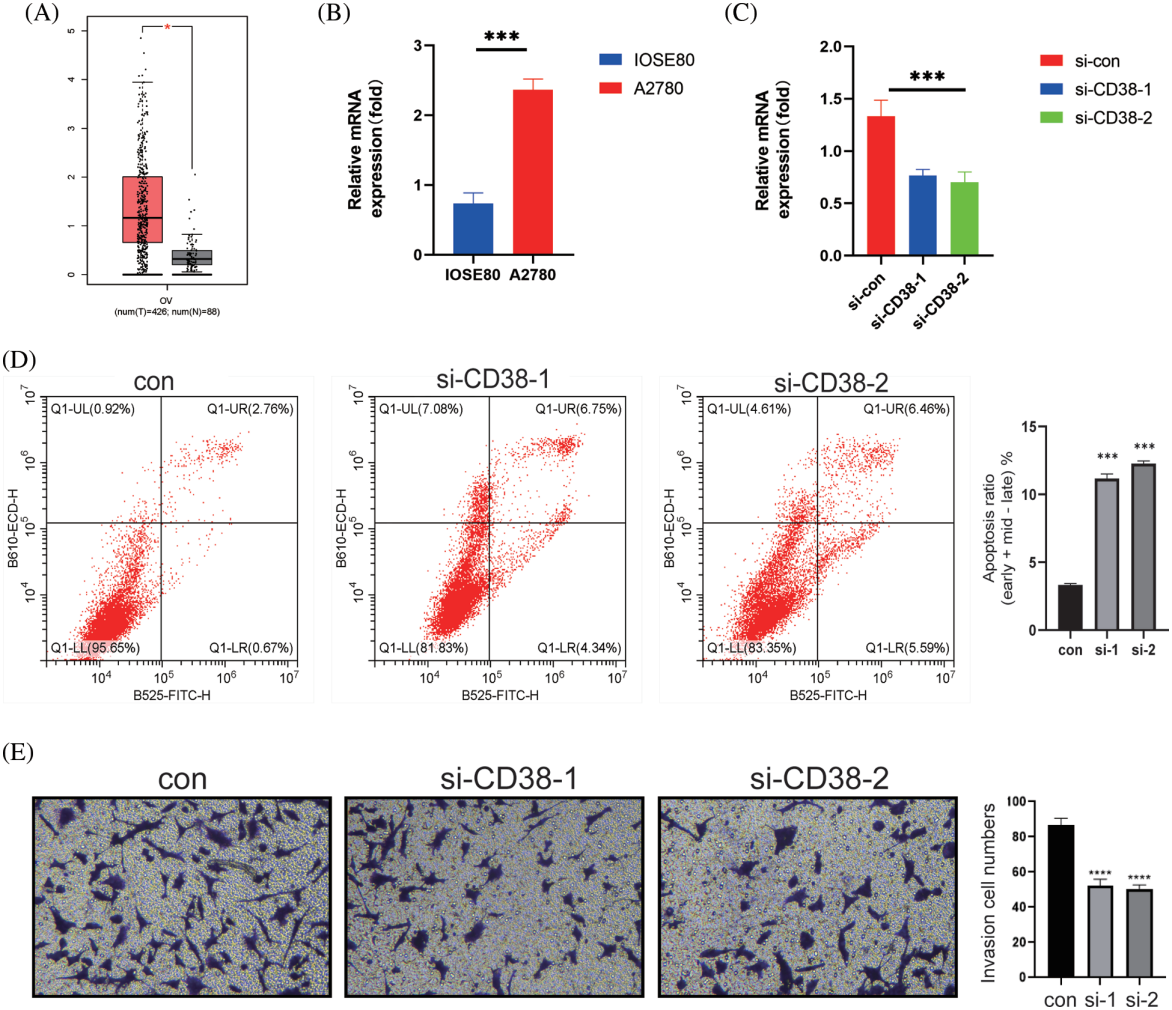
CD38 inhibited the malignant potential of OV cells. (A) Box-line plot showing that *CD38* expression is significantly higher in the OV group. (B) Histogram showing the expression levels of *CD38* gene in normal ovarian cells and OV cells. (C) Histogram showing knockdown of *CD38* gene expression levels in OV cells. (D) Flow cytometry scatter plot showing apoptosis rates of OV cells after *CD38* knockdown. (E) Representative images and histogram of Transwell assay showing invasion ability of OV cells after *CD38* knockdown.

*CD38* was the only model gene that was significantly associated with prognostic indicators in OV patients. In addition, *CD38* expression levels were significantly higher in the OV tissues compared to that in the normal ovarian tissues (Fig. 7A). Consistent with this, the OV cell line A2780 expressed higher levels of *CD38* compared to the normal ovarian cells IOSE80 (Fig. 7B). To further evaluate the biological function of *CD38* in OV, we silenced the gene in A2780 cells. As shown in Fig. 7C, *CD38* was significantly downregulated in the OV cells following gene knockdown. In addition, knocking down *CD38* significantly increased the apoptosis rates in the OV cells (Fig. 7D), and inhibited their invasion ability in the Transwell assay (Fig. 7E). Thus, *CD38* is a potential therapeutic target for OV.

### Identification of candidate drugs for OV

**Figure 8:**
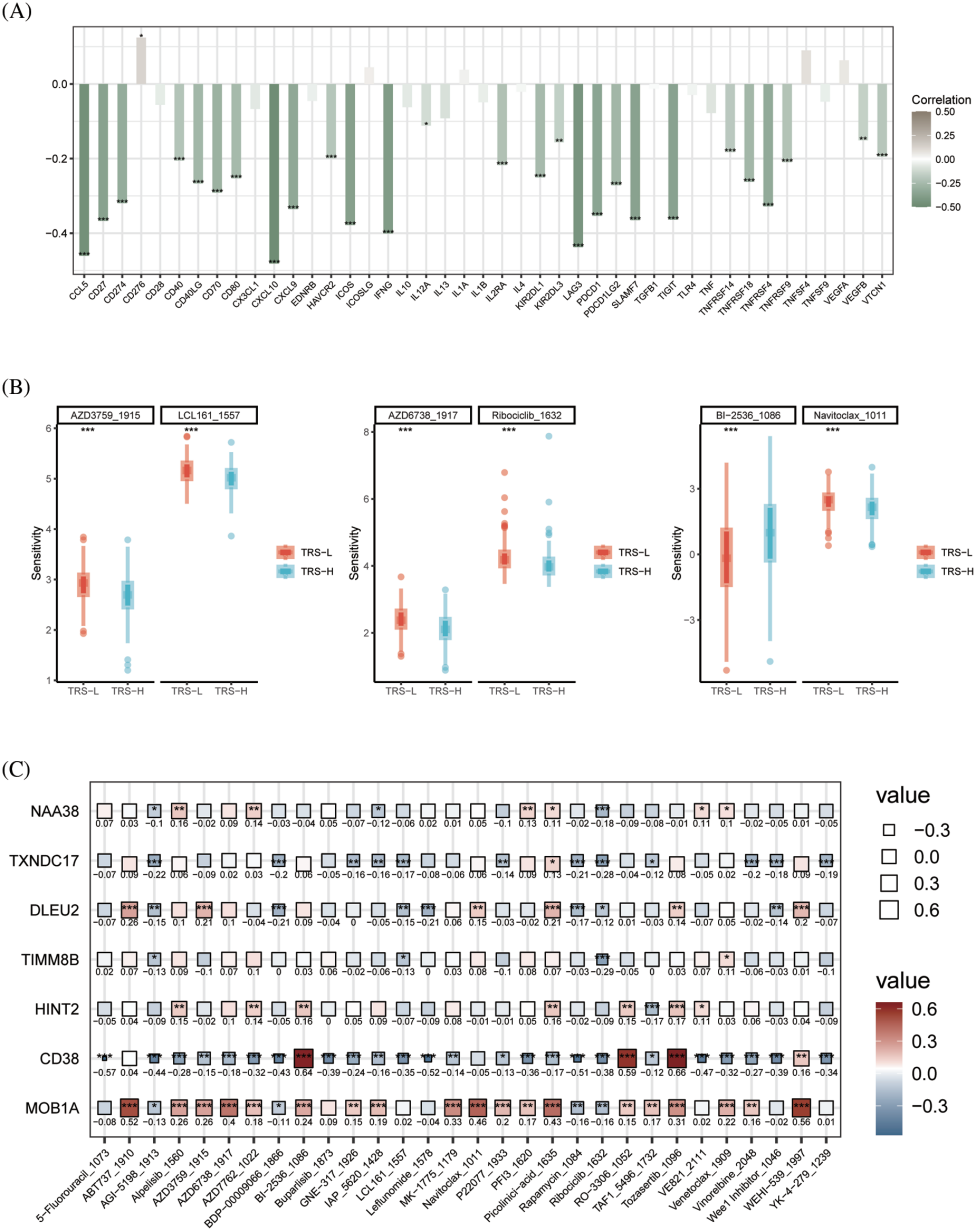
Drug sensitivity analysis. (A) Histogram showing the correlation between the TRS and immune checkpoints. (B) Box plots showing drug sensitivity in the high-risk and low-risk groups. (C) Correlation between each modeled gene and potential drugs.

In addition, we examined the relationship between TRS and the expression of immunomodulators. the TRS was negatively correlated with most immune checkpoints which suggests that TRS may be used to predict the efficacy of immunotherapy (Fig. 8A). To further evaluate the chemosensitivity of OV patients, we assessed the sensitivity scores for each compound. The sensitivity of six drugs was significantly different between the high and low risk groups. The patients in the high-risk group were more sensitive to AZD6738, Ribociclib, AZD3759, LCL161 and Navitoclax, whereas the low-risk group showed greater sensitivity to BI−2536 (Fig. 8B). Furthermore, there was a significant positive correlation between MOB1A and most of these drugs, and CD38 showed correlation with BI−2536, RO−3306 and Tozasertib (Fig. 8C).

## Discussion

OV is one of the deadliest and most aggressive cancers in women, and the incidence has increased in recent years [1]. Given the insidious nature and rapid progression of OV, most patients are not diagnosed until the disease is in the advanced stage [2]. The poor prognosis of OV patients may result from tumor heterogeneity, which restricts prognostic prediction [3]. Currently, surgery and chemoradiotherapy are the most common approaches for treating OV, although the side effects severely reduce the quality of life of the patients [4]. In order to improve treatment outcomes, it is necessary to identify biomarkers for the early diagnosis of OV.

Immunotherapy has emerged as a promising treatment strategy for cancer in recent years, which in turn has spurred the development of immune-related prognostic models. Several studies have reported construction of cancer prognosis models based on genes involved in immune responses, cuproptosis, endoplasmic reticulum stress, angiogenesis, and other processes involved in tumorigenesis, which have shown good prognostic performance, as well as predictive accuracy for immune function [33–37]. Due to considerable intra-tumoral heterogeneity, it is challenging to identify genetic variants through bulk mRNA sequencing. Although the identification of oncogenic drivers by this approach has facilitated the classification of some tumor types, and helped develop new prognostic models, singe cell analysis is crucial to overcome tumor heterogeneity [36,38,39]. ScRNA-seq has been instrumental in identifying the subpopulations within heterogenous tumors, and dissect the molecular mechanisms underlying tumor biology [40,41]. Signature genes screened using single-cell transcriptomic data have been used to establish prognostic models that can not only obviate the limitations of tumor heterogeneity, but also predict prognosis with greater accuracy based on target cell subsets [42–44].

In this study, we identified four distinct T cell sub-populations in ovarian tumors by analyzing the single-cell transcriptional profiles of five OV patients. Pathways related to oxidative phosphorylation and G2M checkpoint were significantly activated in the CD8+ exhausted T cell subset. Oxidative phosphorylation is an integral process in cancer cell growth and development, and a potential therapeutic target [45–47]. In addition, centrosome-associated G2/M checkpoint regulators have also been identified as potential targets for cancer treatment [48,49]. We developed a prognostic model for OV based on seven marker genes for *CD8*+ T cell exhaustion, including *MOB1A*, *TIMM8B*, *CD38*, *HINT2*, *NAA38*, *DLEU2* and *TXNDC17*. The patients were classified as high-risk or low-risk based on the median value of the TRS. Patients in the low-risk group had significantly better prognosis in both the GEO and TCGA cohorts compared to those in the high-risk group. A protein encoded by the *MOB1A* gene is a component of the Hippo signaling pathway, which promotes apoptosis and controls organ size and tumor growth [50,51]. *CD38* encodes a transmembrane glycoprotein that is expressed in several tissues and cells, including those of the immune system. The CD38 protein is a prognostic marker of chronic lymphocytic leukemia and a therapeutic target in multiple myeloma, and has an established role as an immunomodulator in cancer [52]. In addition, CD38 can also predict the prognosis of epithelial OV by enhancing immune infiltration and anti-tumor immunity in the microenvironment [53]. *HINT2* encodes a histidine triplet protein that plays a role in various cancers, and *HINT2* down-regulation can promote colorectal cancer migration and metastasis [54]. NAA38 has not yet been investigated for its specific role in cancer development, but as part of the NatC complex, it inhibits the apoptotic process and is also a potential target [55]. Apart from modulating cell proliferation and invasion, DLEU2 regulates miRNA levels in pancreatic and non-small cell lung cancers [56,57]. TXNDC17 can induce autophagy and promote paclitaxel resistance in OV cells via the tumor necrosis factor-mediated signaling pathways [58].

Immune cell infiltration analysis by MCPcounter and xCell algorithms indicated significant differences between the immune landscapes of the high-risk and low-risk groups. Interestingly, the aDCs, M1 macrophages, M2 macrophages, Th1 cells and Th2 cells were significantly more abundant in high-risk patients than in low-risk patients. Macrophages in the tumor microenvironment can polarize to the pro-tumorigenic M1 phenotype, or the anti-angiogenic and pro-apoptotic M2 phenotype depending on the stimuli [59,60]. OV cells promote polarization of co-cultured macrophages *in vitro*, which may regulate the cytokine profile of the tumor microenvironment [61]. In addition, OV patients with high Th2 mRNA levels have a better prognosis than those with high Th1 mRNA levels [62]. Therefore, we speculate that the difference in prognosis between high-risk and low-risk groups may originate from differences in the immune microenvironment.

Finally, we identified six potential agents that may be effective for OV treatment. Patients in the high-risk group showed greater sensitivity to AZD6738, Ribociclib, AZD3759, LCL161 and Navitoclax, whereas BI-2536 was more suitable for patients in the low-risk group. AZD6738 is an ATR inhibitor, and can overcome chemoresistance in OV cells when used in combination with belitecan [63]. Ribociclib has been effective in recurrent ER-positive OV, and is currently in phase II trials for OV treatment [64]. Therefore, the TRS signature may also help in the selection of optimal drugs for individual OV patients.

The role of T cell exhaustion in ovarian cancer was rarely described in the literature before this paper. Zhou’s team [65] constructed a pan-cancer blueprint for the heterogeneity of T cell exhaustion based on the transcriptome information of 32 tumor types to predict tumor prognosis and therapeutic effect. However, the transcriptome information does not contain the specific information of a single cell line. Therefore, the single-cell transcriptome information was added in this paper to further reveal the mechanism of T cell exhaustion. Based on the work of Professor Zhou’s team, T cell depletion genes associated with ovarian cancer were further explored to construct a model for predicting prognosis and therapeutic effect. Meanwhile, there are some limitations in our study. The TRS was developed and validated using data from public databases, and was not further validated in real-world cohorts. Future research will focus on more robust validation of TRS in multicenter, large cohort data.

## Conclusion

We developed a T cell exhaustion-based gene signature to predict the prognosis of OV patients, and identified *CD38* as a potential therapeutic target.

## Data Availability

The data in this study are from public data set TCGA and GEO. In addition, it also contains the experimental data of our research group.

## Supplementary Materials

**Table S1:**

Gene name
CD38	forward	TCTTGCCCAGACTGGAGAAAGG
	reverse	TGGACCACATCACAGGCAGCTT
GAPDH	forward	GGAGCGAGATCCCTCCAAAAT
	reverse	GGCTGTTGTCATACTTCTCATGG
